# Device and Circuit Analysis of Double Gate Field Effect Transistor with Mono-Layer WS_2_-Channel at Sub-2 nm Technology Node

**DOI:** 10.3390/nano12132299

**Published:** 2022-07-04

**Authors:** Jihun Park, Changho Ra, Jaewon Lim, Jongwook Jeon

**Affiliations:** Department of Electrical and Electronics, Konkuk University, Seoul 05029, Korea; gns6702@konkuk.ac.kr (J.P.); wocwoc3@gmail.com (C.R.); ljw1611@konkuk.ac.kr (J.L.)

**Keywords:** WS_2_, TMD, Sub-2 nm technology, double gate

## Abstract

In this work, WS_2_ was adopted as a channel material among transition metal dichalcogenides (TMD) materials that have recently been in the spotlight, and the circuit power performance (power consumption, operating frequency) of the monolayer WS_2_ field-effect transistor with a double gate structure (DG WS_2_-FET) was analyzed. It was confirmed that the effective capacitance, which is circuit power performance, was greatly changed by the extrinsic capacitance components of DG WS_2_-FET, and the spacer region length (L_SPC_) and dielectric constant (K_SPC_) values of the spacer that could affect the extrinsic capacitance components were analyzed to identify the circuit power performance. As a result, when L_SPC_ is increased by 1.5 nm with the typical spacer material (K_SPC_ = 7.5), increased operating speed (+4.9%) and reduced active power (–6.8%) are expected. In addition, it is expected that the spacer material improvement by developing the low-k spacer from K_SPC_ = 7.5 to K_SPC_ = 2 at typical L_SPC_ = 8 nm can increase the operating speed by 36.8% while maintaining similar active power consumption. Considering back-end-of-line (BEOL), the change in circuit power performance according to wire length was also analyzed. From these results, it can be seen that reducing the capacitance components of the extrinsic region is very important for improving the circuit power performance of the DG WS_2_-FET.

## 1. Introduction

Over the past few decades, semiconductor technology has made progress through scaling down and performance improvements of semiconductors according to Moore’s Law [1] and the Dennard scaling rule [2]. The planar MOSFET process was successfully replaced and commercialized because the so-called FinFET had better electrostatic control. This success of FinFET has led to the 5 nm technology node and is expected to reach beyond the technology node with the introduction of EUV [3,4]. Thanks to these structural changes and the success of FinFET through process optimization, the introduction of a gate-all-around (GAA) structure has recently been actively attempted in academia and industry. Among them, the nanosheet structure is in the spotlight as a strong candidate because it has gate controllability for channels superior to FinFET and more immunity for short channels [5,6,7]. It is expected that scaling due to such a structural change will have a limitation of less than or equal to 3 nm technology node, and a new channel material is attracting attention. Germanium and various III-V material-based channels have better carrier mobility than silicon channels and thus have better electrical properties [8,9]. Additionally, the channel application of two-dimensional materials is actively being studied [10]. Among them, it is noted that a TMD material is thin and thus may effectively reduce a short channel effect and replace silicon due to its excellent interface characteristics and excellent mobility characteristics due to an absence of dangling bond due to Van der Waals bonding [11,12]. In addition, the results of device characteristic analysis through process developments such as contact resistance and doping technology and atomic level analysis have recently been announced [13]. In addition, recently, research on TMD materials has been actively conducted, and research on a FinFET device in which a single-layer TMD material is vertically aligned has been conducted [14,15,16,17,18]. Recently, Z.Ahmed presented DG FET with mono-layer WS_2_ channels and device and circuit power performance that multi-stacked them, showing the possibility of using TMD in sub-2 nm technology node [19]. In this work, the quantitatively analyzed effect of extrinsic components of DG WS_2_-FET on circuit power performance to optimize circuit power performance based on these research results was performed, and a device design guideline for scaling down to improve circuit performance based on DG WS_2_-FET is presented. In addition, the changes in circuit power performance according to various circuit layout types were analyzed.

In this work, based on the DG WS_2_-FET proposed by Z.Ahmed [19], a circuit model library was developed, and device and circuit co-analysis was performed. Through this, the effect of front-end-of-line (FEOL), middle-of-line (MOL), and BEOL on the circuit in DG WS_2_-FET technology is analyzed, and optimization through changes in the performance of the circuit by various K_SPC_ and contacted gate pitch (CGP) by spacer length is analyzed (CGP = L_CH_ + L_CNT_ + 2 L_SPC_). Through this, we present a circuit process development guide for TMD materials that are spotlighted as next-generation materials beyond silicon.

## 2. Device and Circuit Co-Analysis of DG WS_2_-FET

**Figure 1:**
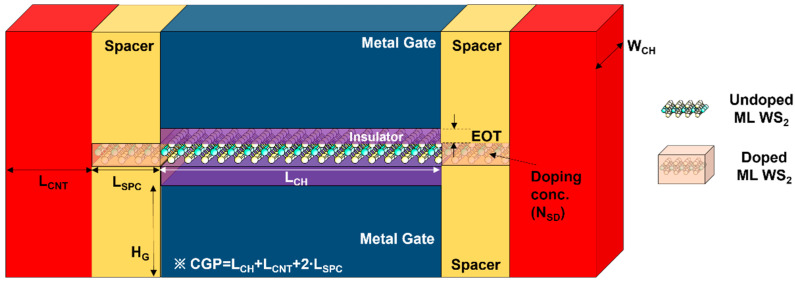
Structure of DG WS_2_-FET used in this work.

The scaling-down technology based on the CGP and metal pitch (MP) becomes the core of the semiconductor scaling technology, enabling low power and high operating speed. However, silicon technology is facing limitations, and TMD continues to scale down beyond its limitations due to its material characteristics. Figure 1 shows the DG WS_2_-FET used in this work. Based on the CGP for the 2 nm technology node [19], CGP by various spacer lengths is presented and summarized as a physical parameter in Table 1. The source/drain extension region below the spacer was considered a heavily doped region with a carrier density (N_SD_) of 1.6 × 10^13^ cm^−^^2^, and the resistance of the extension region is 16 Ω∙μm.

**Figure 2:**
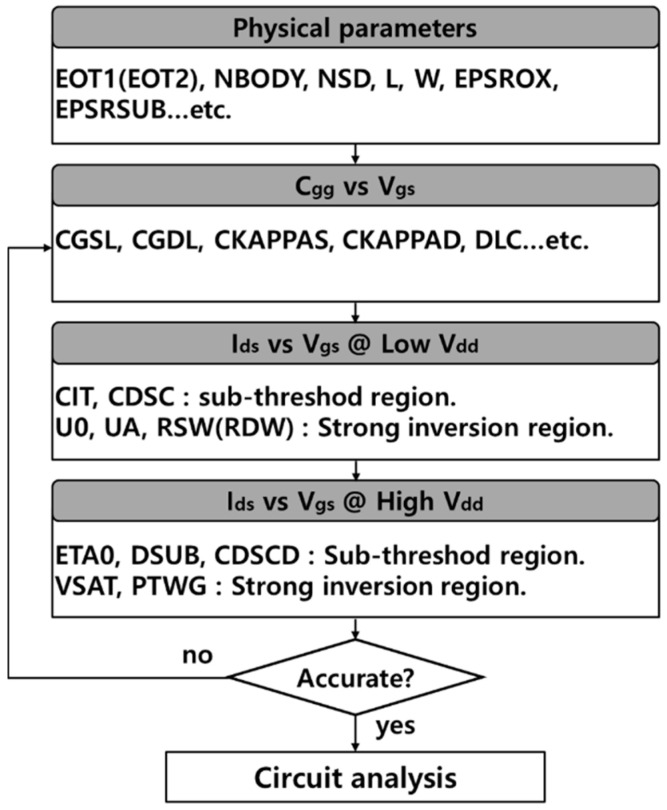
BSIM-IMG model parameter extraction flow used in this work.

**Figure 3:**
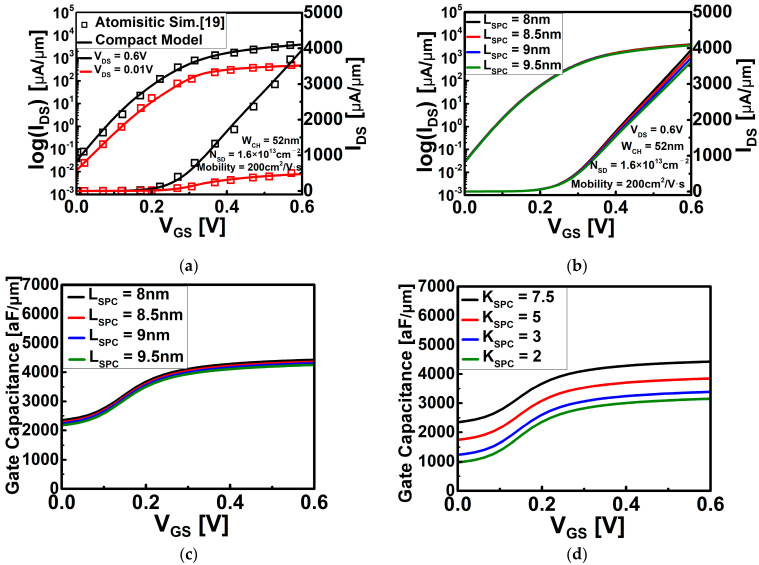
(**a**) I−V transfer curve of DG WS_2_-FET. The black line is when high voltage (V_DS_ = 0.6 V) is applied, and the red line is when the low voltage (V_DS_ = 0.01 V) is applied; (**b**) I−V transfer curve according to L_SPC_ when high voltage applied; (**c**) gate capacitance according to L_SPC_; and (**d**) gate capacitance according to K_SPC_.

The electrical properties of DG WS_2_-FET were obtained using atomistic analysis and the calibrated commercial TCAD simulator. The calibration process of DG WS_2_-FET in Figure 1 was performed using the I-V transfer curve based on the atomistic level simulation of Ref. [19], and through this process, the C-V characteristic curve was obtained to secure the electrical characteristics of DG WS_2_-FET. Note that an effective mobility (= 200 cm^2^/V∙s) of the monolayer WS_2_ channel was estimated in previous work [19] through atomistic calculation, and we take this value in I-V characteristics. Based on the obtained I-V and C-V data, circuit model library generation was performed by using BSIM-IMG [20]. Figure 2 shows the overall BSIM-IMG model parameter extraction flow used in this work. Figure 3a is I-V transfer curve that can confirm the consistency of reference device simulation and performed circuit simulation. The off current (I_OFF_) was the current flowing through the channel when V_GS_ = 0 V and V_DS_ = 0.6 V (supply voltage), and it was targeted at 2 nA. Figure 3b,c are the drain current change and gate capacitance change according to the change of L_SPC_, respectively. As shown in Figure 3b, when the L_SPC_ increases, the current of the DG WS_2_-FET decreases because of the resistance component in the extension.

Region (R_EXT_) increases. This phenomenon is the same as the general phenomenon that appears in devices such as silicon FinFET [21]. However, although the I_ON_/I_OFF_ ratio and subthreshold swing (SS) are noticeably changed in silicon FinFET, there is little I_ON_/I_OFF_ ratio and SS change because the L_SPC_ change is very small in this work (in all cases of L_SPC_ = 8 nm ~ 9.5 nm of DG WS_2_-FET, the I_ON_/I_OFF_ ratio is about 1.33 × 10^5^, and SS is about 69 mV/dec). As L_SPC_ increases in Figure 3c, the gate capacitance decreases because the capacitance component by the gate fringe field (C_EXT_) and the capacitance component between the gate and MOL contact (C_MOL_) are affected by the L_SPC_. That is, it can be seen that L_SPC_ is a key parameter that scales R_EXT_ and C_MOL,_ which are parasitic components excluding the intrinsic components of the device. In addition, it can be expected that there will be a change in the extrinsic component not only in the L_SPC_ but also in the change in the spacer material. Therefore, the influence of the lower dielectric constant of the spacer (K_SPC_) was also investigated. As shown in Figure 3d, the gate capacitance is significantly reduced by reducing K_SPC_ as C_EXT_ and C_MOL_ are reduced by the influence of K_SPC_.

**Figure 4:**
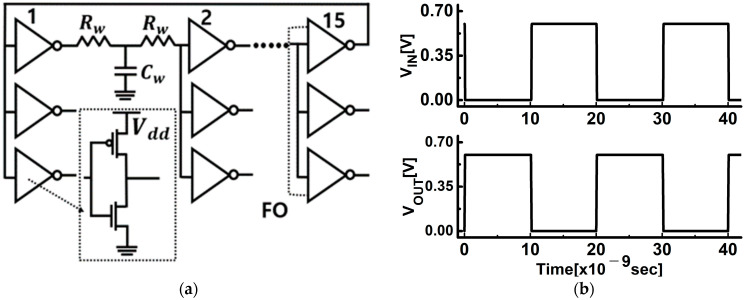
(**a**) Schematic of inverter ring oscillator with fan−out 3, which includes distributed interconnect RC components; (**b**) the transient simulation results of designed inverter ring oscillator.

The circuit simulator and circuit scheme used in this work are Synopsys’ HSPICE and inverter ring-oscillator with fan-out = 3 (FO3 INV RO), respectively, which are widely used in the industry. The FO3 INV RO circuit is depicted in Figure 4a and consists of 15 stages. The R/C component of the BEOL load was attached between the output of one inverter and the input of the next stage. From the INV RO circuit simulation results as shown in Figure 4b, the average signal delay can be extracted to obtain a frequency representing the speed of the operation, and the active dynamic power at the same static power can be extracted.

**Figure 5:**
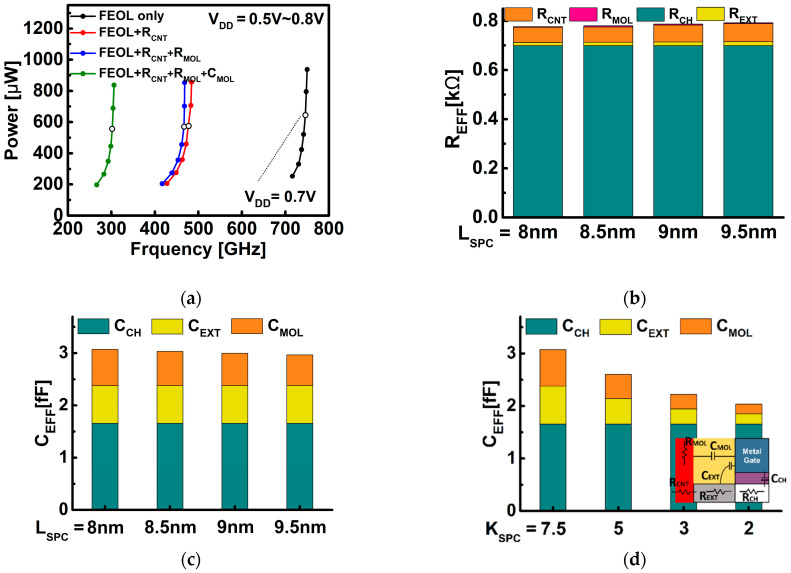
(**a**) Circuit power performance (power consumption, operating frequency) when contact resistance and MOL components are added to the intrinsic channel; (**b**) analysis of results of the effect of various components on circuit using R_EFF_; (**c**) analysis of results of the effect of various components on circuit according to L_SPC_ using C_EFF_; (**d**) analysis of results of the effect of various components on circuit according to K_SPC_ using C_EFF_. The figure inserted in (**d**) shows resistances for channel (R_CH_), extension (R_EXT_), contact (R_CNT_), and MOL (R_MOL_) and capacitances for channel (C_CH_), extension (C_EXT_), and MOL (C_MOL_), respectively.

Figure 5a illustrates the change in circuit power performance when considering contact resistance (R_CNT_) and MOL R/C components (R_MOL_,C_MOL_) with intrinsic channel. A contact resistance of 80 Ω∙μm, the target value of Ref. [19], was adopted. In the developed circuit model, R_CNT_, R_MOL_, and C_MOL_ were considered by attaching these components to both ends of the source and drain of BSIM-IMG model for the DG WS_2_-FET. Based on V_DD_ (supply voltage) = 0.7 V, when R_CNT_ was considered under the same power condition, the operation frequency was decreased by 35.6%, and in addition, considering R_MOL_, it was confirmed that there was a decrease of 2.6%, and when C_MOL_ is added, it is decreased by 35.1%.

The elements that determine the circuit operation characteristics were analyzed using the segmentation technique. This is possible by extracting the operating frequency, the IDDA (active current), and the IDDQ (leakage current) from the inverter ring oscillator circuit.

The operating behavior, and the calculating effective resistance (R_EFF_) and capacitance (C_EFF_), represent the circuit operating speed and power consumption [22]. The circuit characteristics were analyzed by adjusting the WS_2_ channel, contact resistance, and MOL of the circuit model during circuit simulation, and the effects of each component were observed in R_EFF_ and C_EFF_.

The R_CH_ characteristics that vary with the gate voltage of the device are all reflected in the R_EFF_ obtained from the simulation, including the dynamic behavior characteristics of the circuit, which are shown in Figure 5b. The ratio in which the channel and the extension region form the resistance was extracted from V_DS_ = 0.6 V and V_GS_ = 0.6 V under the condition that only FEOL is considered. In Figure 5b, it can be seen that as the L_SPC_ becomes larger, the R_EFF_ also increases. In particular, the effect of the channel, the contact resistance, and the MOL resistance on circuits is almost constant, even if L_SPC_ changes, and it can be seen that R_EXT_ increases. R_EXT_ increased by about 24% as L_SPC_ increased from 8 nm to 9.5 nm. This fact can be explained in Figure 3b as the L_SPC_ increases and the current decreases. In Figure 5c, it can be observed that as L_SPC_ increases, C_MOL_ mainly decreases and the total C_EFF_ decreases. It can be seen from Figure 5d that the C_EFF_ decreases as the K_SPC_ decreases. C_EXT_ and C_MOL_ can be called the parasitic capacitance components, and as the K_SPC_ decreases, it can be seen that the C_EXT_ and C_MOL_ gradually decrease. Through Figure 5b–d, the R_EFF_ can be improved through L_SPC_ scaling, and the importance of the C_EFF_ can be understood through the change of the spacer material.

**Figure 6:**
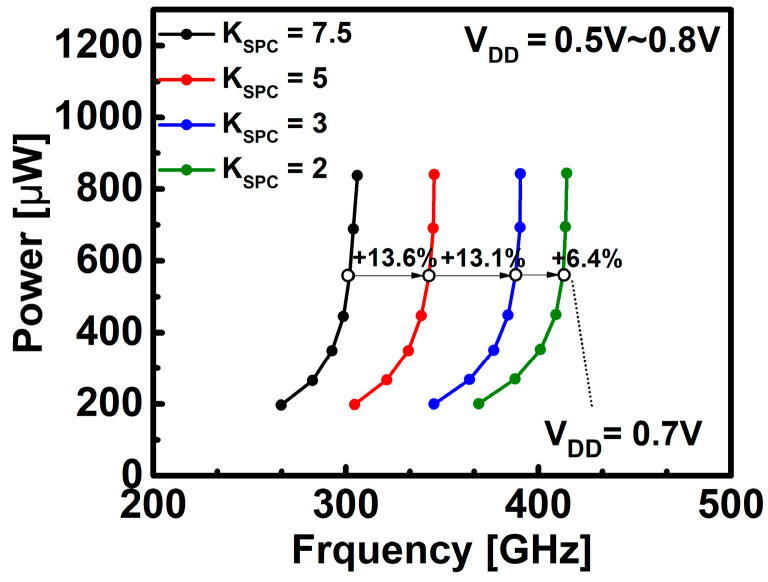
Power versus frequency for DG WS_2_-FET according to K_SPC_.

**Figure 7:**
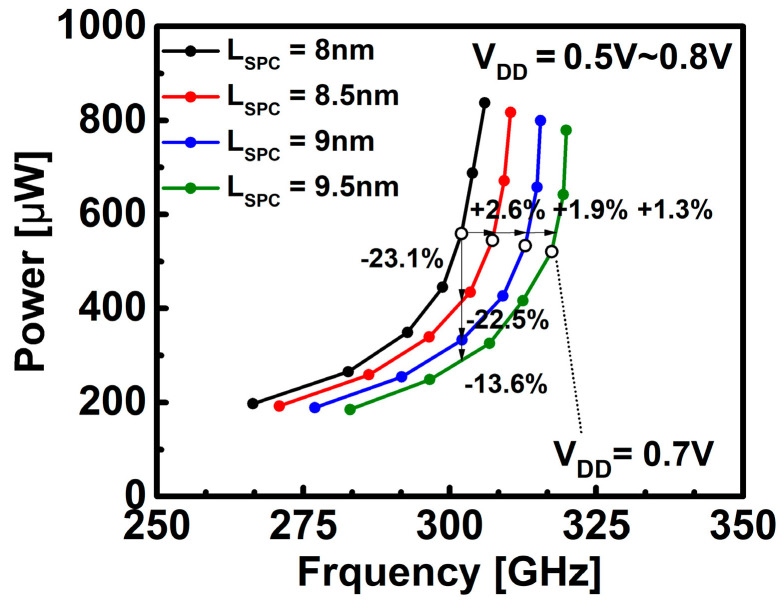
Power versus frequency for DG WS_2_-FET according to L_SPC_.

Figure 6 and Figure 7 show the results of inverter ring oscillator circuit simulation according to the changes in K_SPC_ and L_SPC_. Figure 6 shows that the operating frequency is improved by 13% to 37% at V_DD_ = 0.7 V based on the default K_SPC_ (=7.5). As confirmed in Figure 5c, the operating speed of the circuit was improved through the reduction of the capacitance by the K_SPC_.

As a result of Figure 7, which shows that the performance increases as the L_SPC_ increases, it can be seen that even if the R_EXT_ increases and the overall resistance increases, the performance is improved due to the capacitance component reduced by the L_SPC_. At V_DD_ = 0.7 V, the frequency increases by 2% to 5% and the power decreases by 3% to 7% based on the default L_SPC_ (=8 nm). Since the increased L_SPC_ from the point of view of area scaling is not positive, the improvement of the K_SPC_ is more effective.

**Figure 8:**
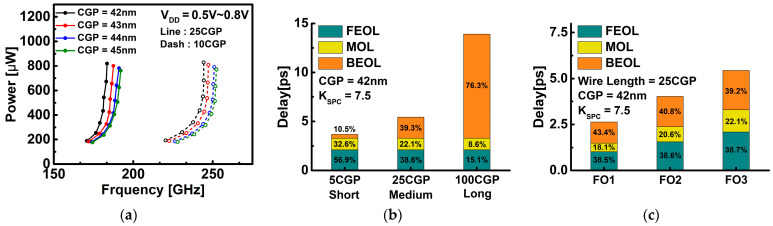
(**a**) Power versus frequency for DG WS_2_-FET according to CGP due to changes in L_SPC_ with BEOL load (K_SPC_ = 7.5). The wire length is 25 CGP, 10 CGP. (**b**) Analysis of delay of designed inverter ring oscillator with BEOL load. The wire length was considered in three cases (5 CGP, 25 CGP, and 100 CGP) in the BEOL load. (**c**) Analysis of delay of designed inverter ring oscillator with BEOL considering fan-out dependency.

Through Figure 8, the effect of the wiring length and BEOL load on the circuit can be analyzed. The wire resistance of the BEOL load was applied as R_W_ = 1447 Ω/μm, and the wire capacitance was applied as C_W_ = 208 aF/μm [23]. As the L_SPC_ changes from 8 nm to 9.5 nm, the CGP changes from 42 nm to 45 nm. Figure 8a shows a power-frequency curve by a BEOL interconnect according to two wiring lengths of 25 CGP and 10 CGP. In each CGP case, it can be seen that the speed change according to the wiring length is 32% to 34%, and the effect of the BEOL component on the circuit is significant. In Figure 8b, the effect of the BEOL load on delay was analyzed by dividing the wiring length into 5 CGP, 25 CGP, and 100 CGP, into short, medium, and long cases, respectively. Based on 25 CGP, the delay decreased by 32% at 5 CGP, and at 100 CGP, the delay increased by 2.5 times. Figure 8c is an analysis of the delay of the circuit according to fan-out dependency when considering the BEOL load. As the fan-out number increases and the total number of inverters in the circuit increases, the delay increases. In addition, it can be seen that not only the delay by the fan-out number increases but also the delay by each component (FEOL, MOL, and BEOL) increases. Figure 8 shows that while the FEOL and MOL processes are of course important, the performance improvements through the BEOL process optimization are essential.

## 3. Conclusions

This work analyzes the effect of performance change through L_SPC_ scaling and K_SPC_ change and the FEOL, MOL, and BEOL components of TMDC FET technology on the circuit based on the previous work using the WS_2_ channel transistor of the double gate structure. In particular, it was confirmed that increasing L_SPC_ is more beneficial to circuit power performance, but there is a trade-off from the viewpoint of area, and it was also seen that the change in K_SPC_ has a great influence on speed improvement. This work confirmed that BEOL optimization is very important, as well as FEOL and MOL, through the effect of the BEOL load by various CGP cases and wiring lengths and the fan-out number on the circuit.

## Figures and Tables

**Table 1:** Key device geometric parameters of DG WS_2_-FET.

Geometric Parameter				
CGP (nm)	42	43	44	45
L_SPC_ (nm)	8	8.5	9	9.5
L_G_ (nm)	14	14	14	14
MP (nm)	16	16	16	16
L_CNT_ (nm)	12	12	12	12
EOT (nm)	0.5	0.5	0.5	0.5
W_CH_ (nm)	52	52	52	52
H_G_ (nm)	20	20	20	20

W_CH_: width of the channel of DG WS_2_-FET.

## Data Availability

Not applicable.
